# Inequalities in pharmacologic treatment of spasticity in Sweden – health economic consequences of closing the treatment gap

**DOI:** 10.1186/s13561-020-0261-7

**Published:** 2020-02-07

**Authors:** Annabelle Forsmark, Linda Rosengren, Per Ertzgaard

**Affiliations:** 1 PharmaLex, Göteborg, Sweden; 2 Ipsen, Kista, Sweden; 3 Department of Rehabilitation Medicine and Department of Medicine and Health Sciences, Linköping University, Linköping, Sweden; 4 Linköping University Hospital, Linköping, Sweden

**Keywords:** Botulinum toxins, Baclofen, Muscle spasticity, Treatment, Access to health care, Health care costs

## Abstract

**Background:**

The Swedish Healthcare Act states that patients should have equal access to healthcare. This study addresses at how this translates to pharmacological treatment of adult spasticity, including injections with botulinum toxin A (BoNT-A) and pumps for intrathecal baclofen (ITB). To address potential economic incentives for treatment differences, the results are also set into a health economic perspective.

Thus, the current study provides a detailed and comprehensive overview for informed decision- and policymaking.

**Methods:**

Botulinum toxin use was retrieved from sales data. Clinical practice regarding mean BoNT-A treatment dose and proportion used for spasticity indication were validated in five county councils, while the number of ITB pumps were mapped for all county councils. Published costs and quality of life data was used for estimating required responder rates for cost-balance or cost-effectiveness.

**Results:**

The proportion of patients treated with BoNT-A varied between 5.8% and 13.6% across healthcare regions, with a mean of 9.2% on a national level. The reported number of ITB pumps per 100,000 inhabitants varied between 3.6 and 14.1 across healthcare regions, with a national mean of 6/100,000.

The estimated incremental cost for reaching treatment equity was EUR 1,976,773 per year for BoNT-A and EUR 3,326,692 for ITB pumps. Based on expected cost-savings, responder rates ranging between 4% and 15% cancelled out the incremental cost for BoNT-A. Assuming no cost-savings, responder rates of 14% or 36% was required for cost-effectiveness.

**Conclusions:**

There is a marked variation in pharmacologic treatment of adult spasticity in Sweden. Overall, the results indicate an underuse of treatment and need for harmonisation of clinical practice. Furthermore, the incremental cost for reaching treatment equity is likely to be offset by spasticity-associated cost-savings.

## Introduction

Spasticity is a common complication of injuries to the central nervous system that often has a negative impact on functioning and can cause severe disability [1]. Physical management is a fundamental part of treatment, often and preferentially integrated with pharmacological treatment for reaching wider rehabilitation aims [2]. The relevant pharmacological regimen is determined by the extent of involvement and the severity of spasticity. Injections with botulinum toxin type A (BoNT-A) is the first-line choice for predominantly focal spasticity [3] and systemic treatment (oral anti-spastic drugs) is warranted with more general involvement. If spasticity is still inadequately controlled, intrathecal baclofen (ITB) is an option, administered through an implanted subcutaneous pump [3]. Two Swedish studies have shown that spasticity in multiple sclerosis and stroke has a significant impact on costs [4, 5], in addition to the impact on daily activities.

Sweden lacks national treatment guidelines regarding management of spasticity, leaving room for local variations in clinical practice. This has been reported in a previous publication, where a marked variation in BoNT-A treatment of adult spasticity was observed [6].

The aim of the current study was to substantiate if the observed differences are persistent and extend to other pharmacological treatment of spasticity.

Thus, in the current study, new data was collected, and the analyses performed with a higher resolution of health care organisation. The study also included a complete national survey of treatment with intrathecal baclofen. Consequently, this is a unique detailed investigation of specialised pharmacological treatment for spasticity with full national coverage, including the potential cost consequences of reaching equal care. Hence, the results are very important for discussions regarding the need of central clinical guidelines for spasticity and to ensure uniform access to treatment.

## Method

### Epidemiology

The epidemiology of spasticity was estimated from published data Table 1.

Primarily, national or Scandinavian references were used, in order to enable translation of the collected BoNT-A use to proportion of treated patients. If more than one suitable reference was identified, a mean from these was used for further calculations. The epidemiology of severe/disabling spasticity in Sweden was computed based on population data for the year 2016 [7], by assuming a uniform regional prevalence. The prevalence of disabling spasticity was used as an approximation of the proportion of spasticity patients eligible for pharmacological treatment.

### BoNT-A

#### Total use

Data on regional use of BoNT-A for the year 2016 was acquired from IQVIA (former IMS Health). Since most pharmacological spasticity treatment in Sweden is performed in public hospitals, the hospital part of BoNT-A was used as an approximation for all spasticity treatment, i.e. BoNT-A sales on prescription was not included.

#### Equivalent units

Potency units for the different BoNT-A products are not interchangeable and there is no defined conversion ratio. For the purpose of this analysis, since there is no defined conversion ratio, the units of the three BoNT-A products used in Sweden (Dysport®, abobotulinumtoxinA; Botox®, onabotulinumtoxinA; Xeomin®, incobotulinumtoxinA) were converted to abobotulinumtoxinA equivalent units based on the publication by Ravenni et al. (abobotulinumtoxinA 500 U, onabotulinumtoxinA 200 U, incobotulinumtoxinA 200 U) [8].

#### Treatment regimen

Nine treatment centers in different county councils representing differences in hospital size, geographical spread and including both university and local hospitals, were contacted for information regarding total number of visits for adult spasticity treatment and total number of BoNT-A units consumed during 2016. Subsequently, an average treatment dose was based on the collected data.

Five of these county councils, in which a single center was responsible for the major part of spasticity treatment, were validated for the proportion of total hospital BoNT-A use specifically for adult spasticity treatment.

Spasticity treatment in the county council Östergötland comprised on average three treatment cycles per patient and year, and this treatment frequency was subsequently used as an approximation for the calculations.

### ITB

For information regarding the total number of ITB pumps, each center performing implantation and/or refill, was contacted during February 2017. All centers in Sweden reported the number of adult patients with ITB for spasticity followed-up at their clinic.

### Costs

#### BoNT-A

In our previous analyses, the cost of closing the estimated treatment gap was calculated [6]. For this analysis, the region with the highest proportion of treated patients was assumed to be closest to an optimal treatment level. For the corresponding calculations in this study, the BoNT-A treatment cost was derived from the Cost Per Patient database using the action code AA026 (electromyography guided injection of botulinum toxin) for the ICD-10 diagnostic codes G811 (spastic hemiparesis), G821 (spastic paraparesis) and G824 (spastic tetraparesis). The reported Cost Per Patient includes drug costs [9].

#### ITB

The cost for ITB delivered via a subcutaneous pump was retrieved from the Southern healthcare region pricelist, for the procedures XO50 (implantation of pump or injection port) and DT028 (refill of implanted pump). The price of baclofen was added. It was assumed that each pump is refilled 3 times annually and that the device lifetime is seven years. Since the pump comes in two sizes (20 or 40 ml), an average volume of 30 ml per refill event was approximated.

#### Spasticity associated costs

Spasticity related costs were derived from published data for patients with multiple sclerosis (MS) [5] and stroke [4]. In the publication regarding MS by Svensson et al., the total cost per year (including healthcare and societal costs) for severe spasticity was 180,759 EUR, while the cost for moderate and mild spasticity was estimated at 136,025 and 75,239 EUR, respectively [5]. Hence, the cost difference between severity grades of spasticity was 60,786 EUR (mild to moderate) and 44,554 EUR (moderate to severe). In the first-year post-stroke, Lundström et al. reported about four times higher costs for patients with spasticity compared to patients with no spasticity. The difference amounted to 62,353 PPP$ (Purchasing Power Parities US dollar, 2003 value) [4], equivalent to around 90,000 EUR in 2017. Thus, as previously reported [6], transition between severity grades of spasticity (defined as responders, as outlined in Table 4) was conservatively associated with an annual cost difference of 45,000 EUR, corresponding to the lowest reported difference in annual cost. The cost includes indirect costs, which were excluded in a sensitivity analysis to reflect the healthcare perspective. Furthermore, the cost also includes botulinum toxin, which has already been taken into account. However, the cost for botulinum toxin as compared to the total cost is negligible and was thus not corrected for.

### Analyses

For this analysis, the proportion of hospital BoNT-A use was validated in those county councils where the reporting centers manage most of the region’s spasticity treatment. The mean proportion of BoNT-A use for spasticity treatment, in these five county councils was 34%.

For simplification, an assumption of equal regional distribution of disabling spasticity was made.

Transition between severity grades of spasticity was associated with an annual cost difference of 45,000 EUR (as described above).

The incremental cost of filling the estimated treatment gap was assessed by assuming that the county council with the highest level of BoNT-A use is closest to an optimal treatment level. In addition, to explore the impact of optimising treatment with BoNT-A on healthcare expenses, the proportion of responders required to balance the incremental cost was estimated. Calculations were based on the difference in cost across spasticity severity grades as described above. However, since there is uncertainty regarding the degree of association between spasticity and costs, the result was also explored in a subset of analyses where the causal relationship between spasticity and costs were varied by decrements of 25%. Hereby, the already conservative reduction in cost associated with transition of spasticity severity (45,000 EUR) was further reduced. In addition, if assuming no cost savings at all, the required responder rate for cost-effectiveness was estimated based on published data and a Willingness to Pay of 52, 000 EUR (corresponding to 500, 000 SEK at the mean exchange rate for 2017; 1 SEK = 9.6 EUR), which has been defined as a moderate cost per quality adjusted life year (QALY) in Sweden. Post-stroke spasticity is defined as a severe condition by the National Board of Health and Welfare, for which a moderate to high cost per QALY is generally accepted. Responder rate estimates for cost-effectiveness was based on two sets of published QALY weights and required number of patients transitioning from severe to moderate spasticity. For simplification, responders were assumed to undergo the transition at first injection and stay in the lower severity state for the rest of the year. These assumptions were considered reasonable based on published data [10, 11].

## Results

### Epidemiology

Epidemiology of disabling spasticity and major underlying conditions were derived from published data as outlined in the Methods section, and a mean prevalence of spasticity per 100,000 inhabitants was calculated (Table 1).

**Table 1:** Epidemiology of spasticity

Condition	Prevalence/100,000	Prevalence disabling spasticity
Stroke	715 [12]	17% [13–15]
CP	215 [16]	23% [17]
MS	190 [18]	33% [19]
TBI	150 [20]	19% [21]
SCI	32 [22, 23]	31% [24, 25]

Mean prevalence of disabling spasticity for all diagnoses = 271/100,000 ^a)^

Abbreviations: *MS* (multiple sclerosis), *CP* (cerebral palsy), *SCI* (spinal cord injury), *TBI* (traumatic brain injury)

^a^Assuming an even distribution in disabling spasticity across healthcare regions

### BoNT-A

#### Total use

BoNT-A use for treating disabling spasticity in adults was set to 34% of the total hospital BoNT-A use, based on the reported mean in the five validated county councils (see Methods section).

#### Treatment regimen

The variation in mean dose per treatment session between the centers was large, with a maximum of 1253 and a minimum of 399 abobotulinumtoxinA equivalent units. The mean treatment dose in all validated centers was 801 abobotulinumtoxinA equivalent units, assuming a mean of three annual treatment sessions per patient. The mean dose, and number of visits per center is shown in Additional file 1: Figure S1.

### Proportion treated patients

The proportion of patients treated with either BoNT-A or ITB was calculated from the total estimated patient population eligible for treatment.

The proportion of treated patients, based on prevalence of disabling spasticity and BoNT-A hospital use, was estimated on the level of county councils as well as for healthcare regions. The variation ranged between 3.9% and 18.8% across county councils and between 5.8% and 13.6% across healthcare regions (Table 2). The total mean proportion of treated patients with disabling spasticity in Sweden as a whole, was 9.2%.

**Table 2:** Incremental cost for regional treatment equity

Healthcare region	Total incremental number of BoNT-A treatment sessions^a^	Total incremental cost per year^b^ (EUR 2017)	Total incremental number of ITB pumps^c^	Total incremental cost per year^d^ (EUR 2017)
Stockholm-Gotl.	946	542,999	250	1,033,523
Örebro-Uppsala	833	656,135	201	833,023
Western	1143	222,295	176	727,773
Southern^e^	–	–	137	567,799
South Eastern	387	477,924	40	164,573
Northern^e^	135	77,420	–	–
Total	3443	1,976,773	804	3,326,692

^a^Assuming highest level reported by the healthcare regions (13.6%) corresponds to optimal level

^b^Based on the mean procedural cost per patient for EMG-guided injection of botulinum toxin for ICD-codes G811, G821 and G824 (~EUR 574). Source: Cost Per Patient database (2017), Swedish Association of Local Authorities and Regions

^c^Assuming highest level reported by the healthcare regions (14.1/100,000) corresponds to optimal level

^d^Based on the total cost for implantation of pump, drug costs and three refills of 30 ml per year

^e^These healthcare regions used as reference for treatment equity of BoNT-A treatment and ITB pumps, respectively. Hence no values for incremental number are assigned

Regarding the number of ITB pumps, the reported number per 100,000 inhabitants varied between 2.1 and 18.8 across county councils, and 3.6 and 14.1 across healthcare regions. The total mean for the whole country was 6/100,000 (Additional file 1: Table S1).

### Regional trend

**Fig. 1:**
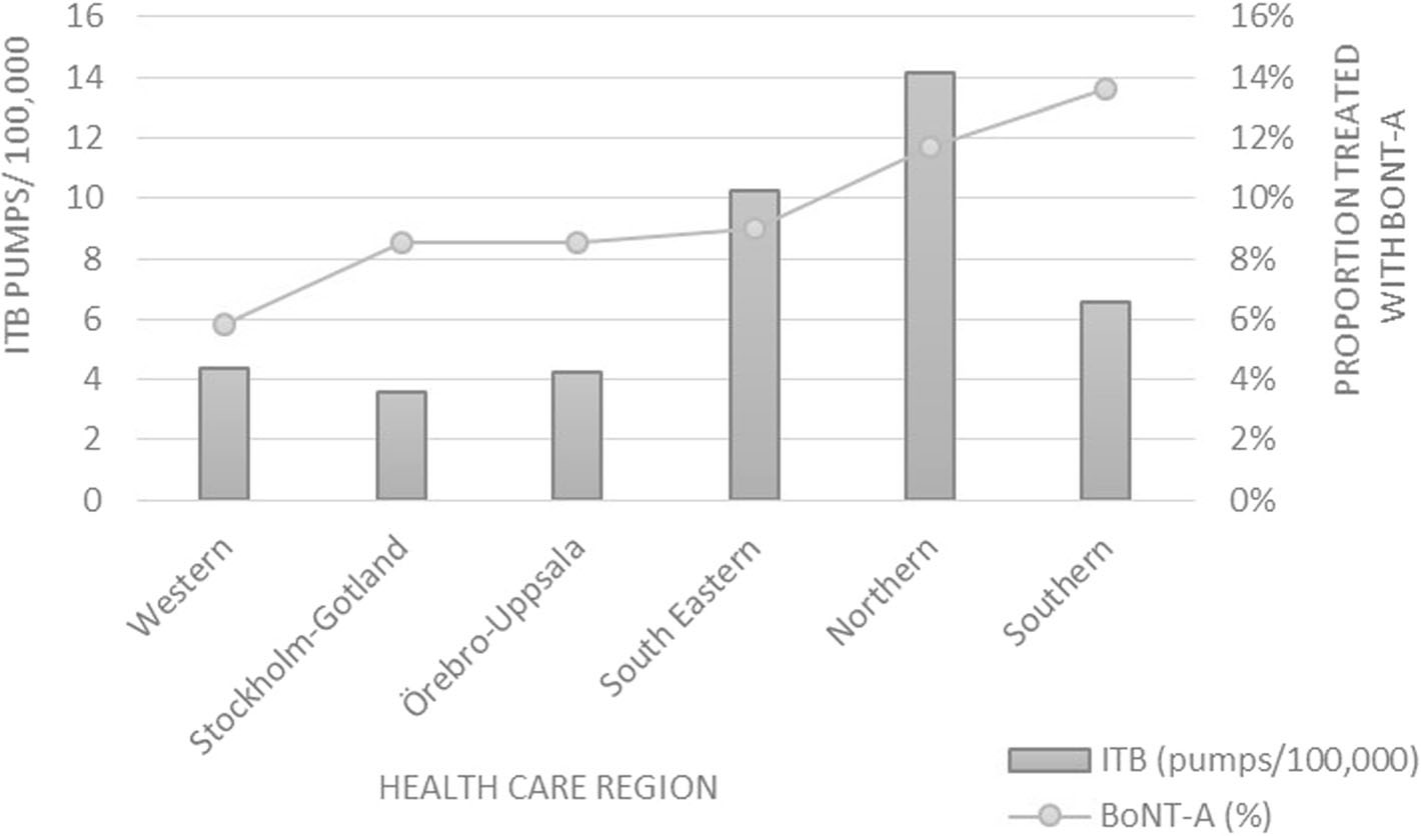
Actual number of ITB pumps per 100,000 inhabitants, and proportion of eligible patients being treated with BoNT-A

The level of BoNT-A and ITB treated patients across the different healthcare regions is illustrated in Fig. 1.

### Trend over time

**Fig. 2:**
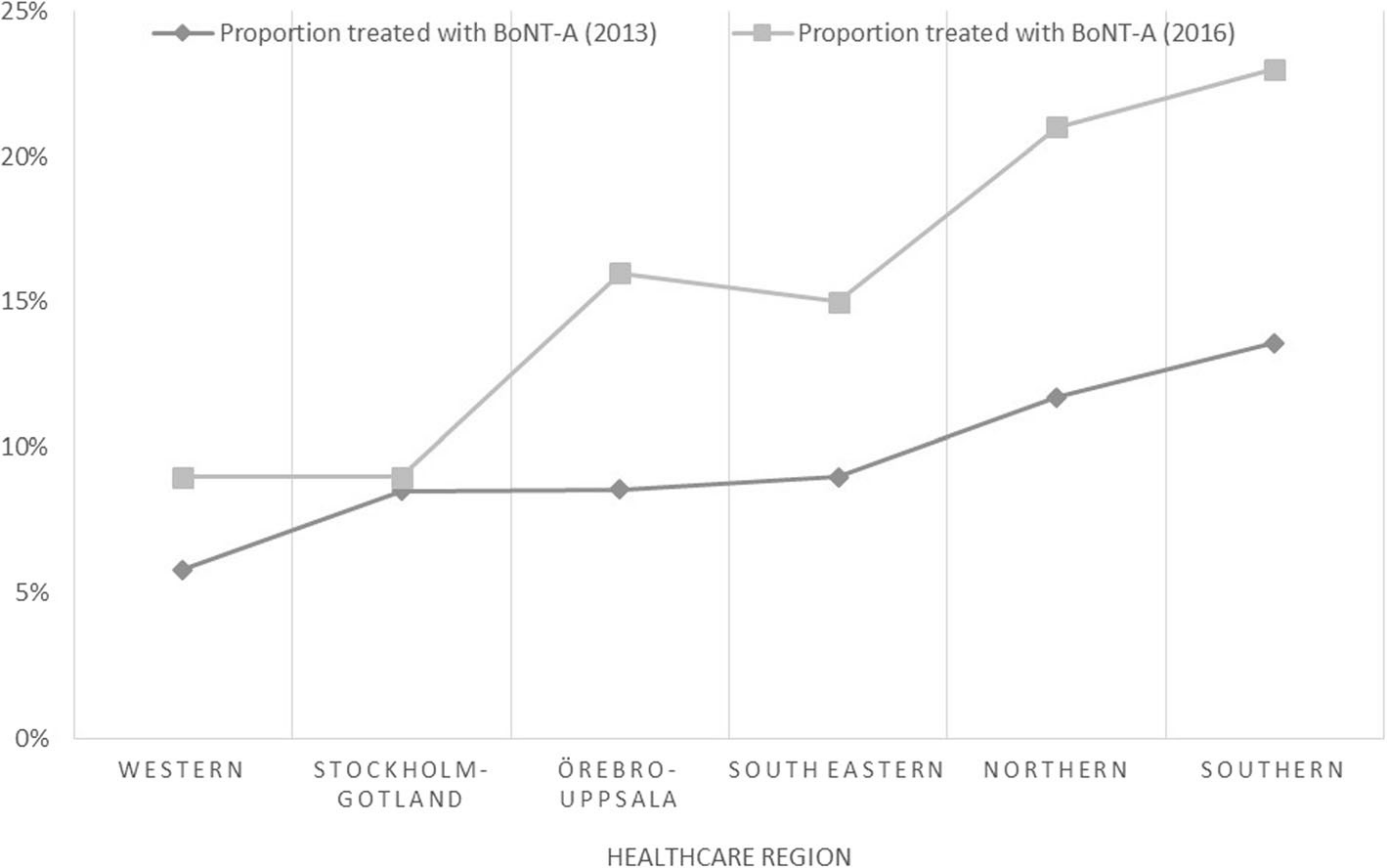
Change in proportion of eligible patient population treated with BoNT-A between 2013 and 2016

Compared to the original study (based on 2013 years data) [6] the trend regarding variations in level of treated patients is virtually the same on the level of healthcare regions. However, the proportion of treated patients is lower overall (Fig. 2).

### Costs

#### BoNT-A

The total incremental number of BoNT-A treatment sessions by region (corresponding to total units consumed divided by the validated mean dose of 801 units) to achieve an equal treatment level as defined in the Methods section was multiplied by the mean cost for EMG-guided injection of BoNT-A (see Method section). The incremental cost was estimated at EUR 1,976,773 per year (Table 2).

#### ITB

The total number of incremental ITB pumps to reach equality were multiplied by the estimated annual cost, as described in the Method section. The incremental cost for attaining the highest reported treatment level of ITB (as reported for the healthcare regions) was estimated to EUR 3,326,692 (Table 2).

### Responder rate for cost balance or cost-effectiveness

In the analysis where the impact of causal relationship between spasticity and cost was explored, the proportion of responders required to balance the incremental treatment cost by savings in spasticity-related costs was low, at about 4%. Assuming a weaker correlation between spasticity and costs, the proportion was still low, but increased to 15% if assuming a level of association of only 25% between spasticity and costs (Table 3). If no consideration to cost-savings were taken, the required responder rate for cost-effectiveness (corresponding to a moderate cost per QALY in Sweden) was 36% if using the utilities reported in Doan et al. [11] for severe and moderate spasticity, respectively. If applying the utilities from Svensson et al. [5] the required responder rate was lower, at 14%.

**Table 3:** Required proportion of BoNT-A treatment responders for cost balance at different degrees of association between spasticity and cost

Scenario	Difference in annual cost between severe and moderate spasticity (EUR)	Required responder rate* for cost balance
Base case scenario^a^	*~ 45,000*	*4%*
Excl. indirect costs	*~ 39,000*	*4%*
Association between spasticity and costs^b^		
*- 75%*	*33,750*	*5%*
*- 50%*	*22,500*	*8%*
*- 25%*	*11,250*	*15%*
Required responder rate* for cost effectiveness^#^		
QALY weights according to Doan et al. [11]		*36%*
QALY weights according to Svensson et al. [5]		*14%*

^a^Base case scenario; 34% of hospital BoNT-A use for spasticity, cost per BoNT-A treatment session = 574 EUR, full causal (100%) relationship between change of spasticity severity grade and associated cost (45,000 EUR)

^b^Effects of decreased influence on the costs (45,000 EUR, see Method section) associated with transition between severity grades of spasticity

^*^In accordance with the study by Doan et al., treatment responders were defined as patients transitioning to a lower level of disability (severe to moderate), as defined by the Disability Assessment Scale (DAS) [11]

^#^based on a Willingness To Pay of ~ 52,000 EUR (corresponding to 500,000 SEK at the mean exchange rate for 2017; 1 SEK = 9.6 EUR), and assuming transition of responders at first injection and subsequent stability for at least a year

## Discussion

In this study, potential treatment inequalities regarding BoNT-A treatment of adult spasticity was assessed. In addition, cost consequences of addressing the reported treatment inequalities was estimated.

An update of references on epidemiology resulted in a marginally higher prevalence of disabling spasticity compared to the previous publication [6]. The current prevalence was estimated at about 271/100,000 inhabitants.

The validation of BoNT-A treatment dose in nine injection centers revealed a large variation, with the highest reported average dose (1253 abobotulinumtoxinA equivalent units) being roughly three times higher than the lowest reported dose (399 abobotulinumtoxinA equivalent units). The wide range of treatment doses for adult spasticity was not associated with the number of patient visits at the different clinics. As the validation was performed in centers from nine different county councils, the large variation indicates differences in local practice. A corresponding large variation in dose has recently also been reported for children with cerebral palsy in Norway, where there was a 2.5-fold difference in maximum dose/kg of BoNT-A across the country’s 21 treatment centers [26].

The results from the current study show marked regional differences regarding BoNT-A spasticity treatment in Sweden. The variation in eligible patients receiving treatment with BoNT-A ranged between 5.8% and 13.6% across healthcare regions and variation was, as expected, greater at the level of county councils, where the proportion ranged from 3.9% to 18.8%. In total, for the entire country, the mean proportion of patients with disabling spasticity treated with BoNT-A was 9.2%.The Norwegian study on children with cerebral palsy from 2019 reported a similar variation, where the proportion treated with BoNT-A ranged from 38% to 80% between the treatment centers with the lowest and highest proportion, respectively [26].

Regarding the number of ITB pumps, the number varied between 2.1 and 18.8 per 100,000 inhabitants across county councils and correspondingly between 3.6 and 14.1 across healthcare regions. To the best of our knowledge, this is the first national epidemiological study on the use of ITB treatment for adult spasticity. These data are essential for resource allocation when organising healthcare. The highest reported number of ~ 18/100,000 in the county councils is close to the estimated steady-state of 20 ITB pumps per 100,000 inhabitants, formulated at a consensus meeting (Nordic expert meeting) in Copenhagen in 2008. However, the total mean for the whole country is comparatively low at 6/100,000 inhabitants.

In comparison to the previous study [6], the levels of regional BoNT-A treatment for spasticity show the same trend. The general treatment level is lower, mainly explained by the up-dated prevalence data, the validated higher treatment dose and the reported lower proportion of hospital BoNT-A use for adult spasticity. However, the results substantiate the regional disparities shown in the previous study and confirm that there is no general trend for an increase in resources allocated for treatment of spasticity.

The cost for levelling out the variation, and thereby increasing the proportion of patients gaining access to treatment, was associated with an incremental cost of EUR 1,976,773 for BoNT-A and EUR 3,326,692 for ITB pumps. Set in perspective, the costs roughly correspond to 0.03% and 0.05% of the total annual healthcare budget in Sweden (2017), respectively. Importantly, irrespective of cost, equal and adequate treatment is desirable and urgent from a patient perspective.

There are some uncertainties regarding the additional cost for addressing the treatment inequalities. First, the prevalence of disabling spasticity has been collected from different sources. Second, the assumption that the highest use of treatment is closest to the optimal is reasonable, but not evidence based. The treatment dose, based on validated BoNT-A use for adult spasticity, from different treatment centers in Sweden, showed a large variation with roughly a three-fold difference between the highest and lowest reported dose. This likely reflects an actual difference in clinical practice across different settings and has an impact on the incremental cost estimate and probably also on treatment outcome, since average doses in some regions are well below the levels used in clinical trials [27, 28]. In addition, the assumption of three treatment sessions per year will in turn ultimately depend on factors such as administered dose, treatment outcome, patient commitment, healthcare organisation and clinical practice. Consequently, these factors will have a great impact on cost.

The proportion of required BoNT-A treatment responders in order to balance the incremental cost of reaching treatment equity is low at ~ 4%. However, the proportion of responders is highly dependent on the causal relationship between spasticity and costs, where a less strong association calls for a greater proportion of responders to balance the extra cost. The highest required proportion of responders in the sensitivity analyses, 15%, is well below previously published levels of treatment responders. Based on BoNT-A treatment of post-stroke upper limb spasticity, over 60% of patients with severe spasticity transitioned to a milder grade of spasticity after the first injection [11]. When comparing to placebo, an absolute difference of 30% in treatment responders (1 point improvement on DAS scale) in favor of BoNT-A has been reported [10]. This is in agreement with the observations in a German multi-center study, where ~ 30% more responders were reported for the bortulinum toxin treatment arm compared to conventional therapy [29]. Hence, the rough estimate of responder rate for cost-effectiveness also indicates a probability of corresponding to a moderate cost per QALY. The result is in line with the stroke guidelines from the National Board of Health and Welfare, where botulinum toxin treatment of spasticity is stated as having a low to moderate cost per QALY. Evidently, without more complex modelling the estimates for cost-effectiveness are unprecise. They are based on immediate and sustained treatment response and ignores potential reversion to the original health state the following year. However, by not including costs and effects from transitions to even lower health states (mild or no spasticity), the uncertainty includes factors working in both directions. Furthermore, by adhering to the lowest reported difference in spasticity-related costs, the calculations are conservative regarding responder rate for cost-balance. Importantly, the cost-balance and cost-effectiveness calculations are also conservative in the sense that neither take simultaneous effects on both costs and quality of life into account.

The large local variation in pharmacological treatment of spasticity corroborates the previous study and are notable in light of the aim of an equal healthcare in Sweden. Furthermore, the same pattern of high variability in dosing of BoNT-A and proportion treated children with spasticity was recently reported from Norway [26]. The reason for this observed variation in treatment of spasticity, which extends from adults [6, this study] to children [26] and includes BoNT-A as well as ITB is not clear. However, as suggested in the previous study [6] and by the authors of the Norwegian study [26], a likely explanation is the lack of evidence-based central guidelines.

This is strongly supported by the recently published outcomes of a program aimed at providing training to health care professionals using BoNT-A for neurological disorders. This large international program was tested during 2012–2017 and included a total of 728 specialists from 51 different countries (~ 71% from Europe). After completing the training, 93% of the attendees thought they had been given new information and that the training would change their daily practice [30]. In summary, there is an evident need of increasing awareness and harmonisation within the field of spasticity care. A suboptimal and unequal access to treatment, neither motivated by scientific nor economic incentives, is unacceptable for this debilitating neurologic condition.

## Conclusions

Taken together, the current study confirms previously reported inequalities in BoNT-A treatment of spasticity, and that it also applies to other pharmacological treatment. Furthermore, clinical practice is shown to differ regarding dosing of BoNT-A. The health economic consequences of closing the reported treatment gap was also estimated in this study, and indicate that there are no economic incentives for restricting pharmacological treatment of spasticity. The emerging explanation of the observed variation seems to be a lack of treatment consensus and up-to date expertise, extending to many countries. Consequently, in order to improve treatment efficiency and equal access, central evidence-based guidelines and dissemination of clinical expertise are needed.

## Supplementary information

**Additional file 1: Figure S1.** Mean treatment dose (converted to abobotulinumtoxinA equivalent units) reported per contacted center (2016). **Table S1.** Geographical variation in spasticity treatment

## Data Availability

All data supporting the results and conclusions in this study are available on request to the authors.

## Abbreviations

BoNT-A: Botulinum toxin A
CP: Cerebral Palsy
DAS: Disability Assessment Scale
EUR: Euros
ITB: Intrathecal baclofen
MS: Multiple Sclerosis
QALY: Quality Adjusted Life Year
SCI: Spinal Cord Injury
SEK: Swedish Crowns
TBI: Traumatic Brain Injury
