# Supplementary material for: Inequalities in pharmacologic treatment of spasticity in Sweden – health economic consequences of closing the treatment gap
Source: Health Econ Rev. 2020 Feb 7;10:4. doi: 10.1186/s13561-020-0261-7 (PMC7006187; doi:10.1186/s13561-020-0261-7)
Supplement: Supplementary file 1 — Additional file 1: Figure S1. Mean treatment dose (converted to abobotulinumtoxinA equivalent units) reported per contacted center (2016). Table S1. Geographical variation in spasticity treatment [file 13561_2020_261_MOESM1_ESM.docx]

**Supplement. *Inequalities in pharmacologic treatment of spasticity in Sweden – cost consequences of closing the treatment gap***

**Figure S1.** Mean treatment dose (converted to abobotulinumtoxinA equivalent units) reported per contacted center (2016).

**Table S1.** Geographical variation in spasticity treatment

| Health care region  *- county council* | Population | Patients treated with BoNT-A /100,000 ^a)^ | Proportion of patients with disabling spasticity treated with BoNT-A ^b)^ | Patients treated with ITB /100,000 ^c)^ | Proportion of patients with disabling spasticity treated with ITB ^b)^ | |
| --- | --- | --- | --- | --- | --- | --- |
| Stockholm/Gotland | **2,318,000** | **23.1** | **8.5%** | **3.6** | | **1.3%** |
| *- Stockholm/Gotland* | *2,318,000* | *22.8* | *8.4%* | *3.6* | | *1.3%* |
| Örebro/Uppsala | **2,052,000** | **23.2** | *8.6%* | **4.2** | | **1.6%** |
| *- Uppsala* | *359,000* | *42.8* | *15.8%* | *6.2* | | *2.3%* |
| *- Örebro* | *294,000* | *17.9* | *6.6%* | *4.5* | | *1.6%* |
| *- Dalarna* | *283,000* | *17.2* | *6.3%* | *4.3* | | *1.6%* |
| *- Sörmland* | *287,000* | *17.0* | *6.3%* | *2.5* | | *0.9%* |
| *- Västmanland* | *267,000* | *32.3* | *11.9%* | *2.6* | | *1.0%* |
| *- Värmland* | *278,000* | *14.9* | *5.5%* | *5.8* | | *2.1%* |
| *- Gävleborg* | *284,000* | *14.0* | *5.2%* | *3.2* | | *1.2%* |
| Southern | **1,828,500** | **36.9** | **13.6%** | **6.6** | | **2.4%** |
| *- Halland (Southern part)* | *159,500* | *16.8* | *6.2%* | *4.8* | | *1.8%* |
| *- Blekinge* | *158,000* | *19.4* | *7.2%* | *11.5* | | *4.3%* |
| *- Kronoberg* | *194,000* | *27.1* | *10.0%* | *2.1* | | *0.8%* |
| *- Skåne* | *1,317,000* | *42.3* | *15.6%* | *6.8* | | *2.5%* |
| Western | **1,825,500** | **15.8** | **5.8%** | **4.4** | | **1.6%** |
| *- Västra Götaland* | *1,666,000* | *15.5* | *5.7%* | *4.4* | | *1.6%* |
| *- Halland (Northern part)* | *159,500* | *16.8* | *6.2%* | *4.8* | | *1.8%* |
| South Eastern | **1,043,000** | **24.3** | *9.0%* | **10.3** | | **3.8%** |
| *- Östergötland* | *451,000* | *34.1* | *12.6%* | *13.9* | | *5.1%* |
| *- Jönköpimg* | *352,000* | *10.5* | *3.9%* | *8.9* | | *3.3%* |
| *- Kalmar* | *240,000* | *25.1* | *9.3%* | *5.5* | | *2.0%* |
| Northern | **908,000** | **31.8** | **11.7%** | **14.1** | | **5.2%** |
| *- Norrbotten* | *250,000* | *18.3* | *6.8%* | *18.8* | | *6.9%* |
| *- Jämtland* | *128,000* | *51.1* | *18.8%* | *5.5* | | *2.0%* |
| *- Västerbotten* | *265,000* | *35.6* | *13.1%* | *17.8* | | *6.6%* |
| *- Västernorrland* | *265,000* | *28.5* | *10.5%* | *9.8* | | *3.6%* |
| *Mean whole country* | ***9,975,000*** | ***24.9*** | ***9.2%*** | ***6.0*** | | **2.2%** |

^a)^ Based on hospital sale of BoNT-A from IMS Health 2016, a mean of 3 treatment sessions/year, 34 % proportion of BoNT-A hospital use for adult spasticity, 801 abobotulinumtoxinA equivalent units/treatment session, and the estimated patient population eligible for pharmacological treatment of spasticity (see Methods section in main document).

^b)^ Based on the estimated patient population eligible for pharmacological treatment of spasticity (see Methods section).

^c)^ Based on total number of ITB pumps as reported per county council.
